# Conventional electroencephalography for accurate assessment of brain maturation in preterm infants following perinatal inflammation

**DOI:** 10.1038/s41390-022-02185-7

**Published:** 2022-07-19

**Authors:** Antoine Giraud, Carol M. Stephens, Geraldine B. Boylan, Brian H. Walsh

**Affiliations:** 1 INFANT Research Centre, University College Cork, Cork, Ireland; 2 INSERM, U1059 SAINBIOSE, Université Jean Monnet, Saint-Étienne, France; 3 Department of Paediatrics and Child Health, University College Cork, Cork, Ireland; 4 Department of Neonatology, Cork University Maternity Hospital, Cork, Ireland

We read with great enthusiasm the commentary article from Professor Mark S. Scher,^1^ in response to our systematic review summarising the association between perinatal inflammation exposure and electroencephalography (EEG) features in preterm infants.^2^

Perinatal inflammation exposure represents an additional risk factor for impaired developmental trajectory in preterm infants.^3^ This additional risk is not only due to the association of perinatal inflammation with cerebral haemorrhage and white matter injury,^4^ but also to the neuronal toxicity of supra-physiological serum concentrations of interleukin-1 beta observed in this situation.^5^ Even in preterm children without severe neonatal brain injury, perinatal inflammation remains independently associated with decreased motor and social abilities at 30 months of corrected age.^3^

EEG is the primary tool for functional evaluation of brain activity in preterm infants hospitalised within the neonatal unit. Serial longitudinal EEG recordings before term age can accurately assess brain maturation in preterm infants, and identify infants with an unfavourable early developmental trajectory.^6^ This identification is crucial to provide individualised early developmental intervention programmes for these infants. Such programmes have been shown to improve motor and cognitive outcomes in preterm children.^7^

We agree that amplitude-integrated EEG (aEEG) is currently a useful tool to supervise the brain activity of critically ill preterm infants hospitalised in neonatal units that do not have 24/7 access to conventional EEG. However, aEEG provides a limited assessment compared to conventional EEG, particularly in very preterm neonates who do not demonstrate distinguishable sleep-wake cycling before 29 weeks of gestational age.^8^ Beyond the estimation of discontinuity and amplitude offered by aEEG evaluation, conventional multi-channel EEG provides key information about other network-based brain activity, such as synchrony, frequency, and reactivity.^9^ It also delivers crucial information on the dynamics of transient endogenous generators occurring through the complex process of brain maturation.^9^ The two studies included in our systematic review were limited to aEEG analysis and reported inconsistent findings associated with perinatal inflammation exposure.^2^ Nevertheless, perinatal inflammation led to a modification of EEG frequencies in most of the preclinical studies assessing its effect on foetal sheep EEG, underlying the importance of the quantitative analysis of conventional EEG in the assessment of early brain maturation.^2^

Conventional multi-channel EEG is essential to assess brain maturation in preterm infants, especially in those exposed to additional developmental risk factors such as perinatal inflammation. The need for a specialist to apply and interpret conventional multi-channel EEG has often been seen as an impediment to its widespread use within the neonatal unit. However, rather than simplifying the process by utilising fewer channels with less information, we believe that modern engineering solutions will allow the widespread introduction of conventional multi-channel EEG in neonatal units. There have been tremendous recent advances in the development of quantitative analysis and machine learning to develop automated algorithms for conventional multi-channel EEG analysis; such automatisation will soon assist in the further introduction of conventional multi-channel EEG beyond specialist centres.^10^
